# Constituent Year Effects and Performance in Alpine Skiing Junior World Championships

**DOI:** 10.3390/sports11080155

**Published:** 2023-08-16

**Authors:** Øyvind Bjerke, Håvard Lorås, Arve Vorland Pedersen

**Affiliations:** 1 Department of Teacher Education, Norwegian University of Science and Technology, 7491 Trondheim, Norway; 2 Department of Neuromedicine and Movement Science, Norwegian University of Science and Technology, 7030 Trondheim, Norway

**Keywords:** constituent year effect, relative age, relative development effects, birth year effect

## Abstract

This study examines constituent year effect (CYE) and race performance among junior alpine skiers in the World Championships. In various junior age cohorts competing together, variation in skiing performance can be expected not only due to practice load and experience but also due to inter-individual differences in physical and psychological maturation. Within a one-year cohort, this effect has been referred to as the birth month effect or the relative age effect (RAE). In cohorts with multiple age bands, the effect is termed the constituent year effect (CYE). The CYE works in principle as the RAE but can function as a magnifying lens of the development within a larger multi-year cohort. The results of the current study indicate that CYEs are present among junior alpine skier performance in the junior World Championships. The magnitude of the constituent year effect is greater in speed events (i.e., downhill and super-G) than in technical events (i.e., slalom and giant slalom), and greater among male skiers compared to female skiers. The findings are discussed in relation to previous research on relative age effects more generally and within the sport context specifically.

## 1. Introduction

Both the time and date of birth can play an important role in people’s lives. In sports competitions, athletes are typically placed in groups according to their time of birth, where the most typical category is annual grouping due to the chronological age [1]. This means that athletes born between the cut-off dates of January 1st and December 31st in the same calendar year are assigned to the same group. The intention of this grouping is to provide equal and fair competitions by trying to reduce the maturational differences between athletes competing together. However, this annual grouping leads to an almost one-year age difference between athletes born early and those born late in the same year, and this difference in age is referred to as relative age [2]. The potential differences within a one-year cohort of athletes are well established, and the consequence of this grouping is known as the relative age effect (RAE) [3,4].

Relative age effects are found in a lot of contexts, and the effect varies with several variables. One of the factors is body mass, with a more pronounced RAE in sports, where size, strength and weight are advantageous [5,6]. Typical sports are ice hockey [7], rugby [8], soccer [9] and basketball [10]. In sports where weight and size are a disadvantage, the RAE is absent or even reversed, as documented in sports like gymnastics [6] or ski jumping [11]. Also, in sports that are largely dependent on technical (motor) skills, the RAE is less likely to be found [12]. Another variable coupled with the RAE is the performance level, where a high performance level is associated with a more pronounced effect [1]. Even within one single sport, there seem to be variations in RAE. This can be exemplified in handball and soccer, where RAE can vary according to player position, e.g., certain somatotypes are associated with player positions [13]. Similar variations are found in alpine skiing, where the effect is identified in speed events but not in technical events [14]. Gender is also a variable, as it is more pronounced among male athletes compared to female athletes within the same sport [15]. However, in a review by Smith et al. [16], it is argued that the effects among female competitors still exist, but they are masked due to smaller groups, less selection pressure, lighter competition level and differences in physical demands.

When growing older, athletes are usually allocated to age categories that contain several increments or years in the same age category, e.g., in junior sports and in Masters sports, the age span varies from 2 to 5 years. In these age bands, the effects of the relative age work over a longer time span, leading to what is known as the constituent year effect (CYE) [2]. The impact of an athlete’s constituent year increases with the age range in the multi-year age band, meaning that the effect is greater among a 5-year age band than among a two-year age band. A CYE works similarly to RAE in that the effects are more pronounced among children and adolescents in that the youngest athletes in the multi-year age band are more likely to succeed. At a certain age, there will be a shift in the effect, as the effect is found to be inverse among athletes in Masters sport. In studies by Medic et al. [17] and Medic et al. [18], the probability of participating in the US championships in masters swimming and track and field was higher if the athletes were in the first and second year and lower if they were in their fourth or fifth year in the five-year age band category. This switch, in effect, can be explained by peak performance normally occurring around 27 years, albeit depending on the sport characteristics [19].

The advantages of being stronger and heavier than your peers in the same multi-age cohort are reported, especially in sports that require high demands in terms of physical properties [20]. Thus, it has been argued that the CYE works similarly to RAE, only stronger because the relative age in the cohort works over a longer time period [21]. Thus, the CYE will work as a magnifying lens on the RAE, as it makes any change or variation more visible. In the paper by Bjerke et al. [21], who studied junior alpine skiers participating in the Junior World Championship (JWC), it was found that participation varied with age across the 5-year age span. In fact, participation varied almost linearly with age, with significantly fewer younger skiers (17 to 18 years) competing in the JWC compared to older skiers (20 and 21 years). Additionally, in that study, participation varied with the speed of the events: the higher the speed, the older the athletes. The effect of speed varied with gender in that it was evident two years later for male skiers as compared to female skiers. Most importantly, the results from Bjerke et al. [21] suggest that relative age may not be the important variable for explaining age-related differences in performance since the effect of age is not equal for the different events and also varies with sex. Rather, it seems that the RAE may act as a proxy for relative development, which may explain both the variations in speed and the differences between sexes due to differences in puberty offset. Hence, it would be more correct to replace the term relative age effect with relative development effect.

The purpose of the current study was to further examine the potential relationship between race performance and the age of participants in the alpine skiing Junior World Championships. Not least because Bjerke et al. [21] did not measure actual performance but used participation as a proxy. The present study aimed to investigate whether that proxy was indeed a good representation of actual performance and, thus, whether the results and conclusions would hold. Based upon the presented considerations, it was hypothesised that (1) the CYE is closely related to the skiers’ performance in that the older the skiers are within the five-year age span, the better their results are. (2) The CYE is more pronounced among male skiers compared to female skiers, and (3) the relationship between CYE and performance is stronger, if not linearly related to speed, in the speed events (Downhill and Super-G).

## 2. Materials and Methods

### 2.1. Study Sample

The male and female alpine skiers participating in the FIS Junior World Ski Championship in alpine skiing in Sochi 2016 (N = 705), Åre 2017 (N = 746), and Davos 2018 (N = 596) were selected for the present study. This comprised an overall sample of 1188 male skiers and 859 female skiers within an age range of 17–21 years at the time of competition, with birth years between 1995 and 2001 (see Table 1).

### 2.2. Study Variables

Data were obtained from the Fédération Internationale de Ski (FIS) website (www.fis-ski.com) (accessed on 18 June 2018) [22] and included the skiers’ year of birth, sex, results in competitions (completed first run, second run, and final ranking in the competition), and events (slalom, giant slalom, super-G, and downhill). The year of birth was used to compute the age of each skier at the time of competition.

A skier’s performance was initially measured by completing or not completing a race. The results did not distinguish whether a skier failed in the first or second run or the reason for not completing, e.g., disqualified, did not start the second run, or did not finish. The speed events (super g and downhill) include only one run, whereas there are two separate runs in the technical events (slalom and giant slalom). A second measurement of performance is the final ranking in the race, where the winner was ranked 1, second place was ranked two, and so on. The mean rank for each event was calculated and compared across age groups and sex.

### 2.3. Statistical Analysis

The performance data demonstrated considerable positive skewedness and non-normal distribution according to a significant Kolmogorov–Smirnov test across both male and female sub-samples. Thus, statistical analyses proceeded with non-parametric approaches. To investigate whether there was a statistically significant trend across age in competition and overall race performance, Jonckheere–Terpstra tests for ordered alternatives were applied. To assess potential differences between age cohorts in completed/not-completed races, Odds Ratios (ORs) and 95% confidence intervals (95% CIs) were calculated according to these categorisations. Predictive Analytics Software (PASW, IBM, NY, US; previously SPSS) Version 27.0 was used for all statistical procedures, with *p* < 0.05 as the statistical significance criterion.

## 3. Results

### 3.1. Male Junior Alpine Skiers

#### 3.1.1. Completed Races

Overall, there was a mean completion rate among male skiers of 56.4% across all the age ranges and across all events (see Table 2). Among all events and through ages 18–21, the mean completion rate varied from 53.3 to 58.5% except for 17-year-old skiers, where 42.6% of the skiers completed the race. At the same time, there were fewer skiers who participated as 17-year-olds (N = 54) than those in the age categories 20 and 21-year-old (N = 407 and N = 383, respectively). This is shown in the pairwise comparisons in Table 3 with the statistics for Odds Ratio, with a significantly different overall ratio between 17-year-old skiers and 20- and 21-year-old skiers.

The percentage of skiers who completed a race varied across events, as shown in Table 2. The mean completion rate for slalom was 30% across all ages, and this rate increased with the speed of the event, ending up with a mean completion rate of around 92% in downhill. The lowest completion rate was among the 17- and 18-year-old skiers in slalom, with a completion percentage of 25.0 and 23.3, respectively. This indicates that 75% of the skiers did not succeed in completing the race, either by falling, hooking, or being disqualified in one of the two runs. In downhill, the mean completion rate was above 90% across all ages, with only 10% not finishing the race. In the speed events (super G and downhill), there were also fewer skiers starting than in slalom and giant slalom. In the pairwise comparisons, the ORs, no statistically significant differences were found when the results were separated by event and age.

#### 3.1.2. Performance in Competition

**Figure 1:**
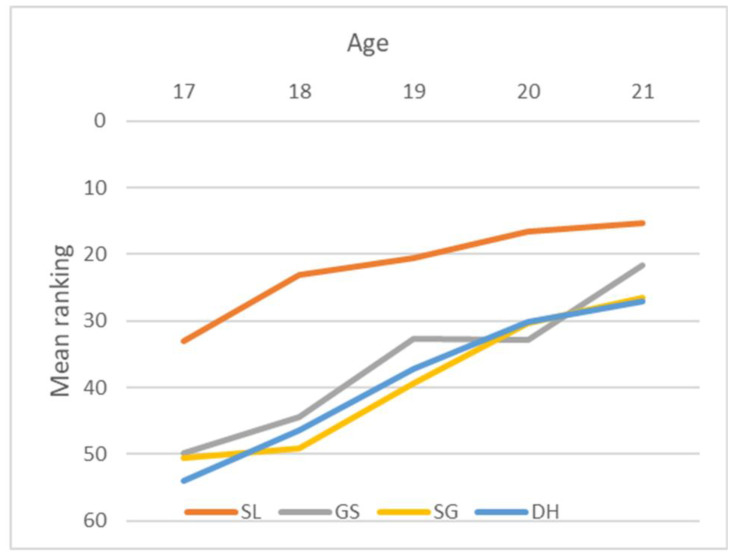
Increase in mean ranking across age category and events in male skiers. A lower ranking indicates better performance. (SL = slalom; GS = Giant slalom; SG = Super G; DH = downhill).

The performance increased with age, as shown in Table 4 and Figure 1, with a declining mean ranking with age. The rate of decrease was almost similar for all events, following the same declining mean rank for giant slalom, super-G, and downhill. Also, the rate of decrease for slalom was similar to the other events, but because of a smaller N of skiers that completed the race, the mean rank for each event was lower at all ages. In slalom, the N was 108 skiers, whilst around N 190 for the other events. The Jonckheere–Terpstra test for order alternatives showed a statistically significant trend (*p*-value < 0.005) of lower mean rank with age in male skiers in all events, as shown in Table 5.

### 3.2. Female Junior Alpine Skiers

#### 3.2.1. Completed Races

The completion rate in all events and all age categories for female skiers was 63.8%, as shown in Table 6. Across ages, the completion rate varied from 58.5% to 66.3%. The mean completion rate across ages in the events increased with speed, ending up at 93.8% downhill. However, there were greater variations in the descriptive completion rate among female skiers than for males. Also, for the female skiers, the lowest completion rate was also found in slalom, but the deviation around the mean was lower than among male skiers. The mean completion rate downhill was 93.8%. Also, among females, the number of skiers starting downhill was less than in the other events. In the pairwise comparisons with the statistics for the Odds Ratio, no significant differences were found among the female skiers (see Table 7).

#### 3.2.2. Performance in Competition

**Figure 2:**
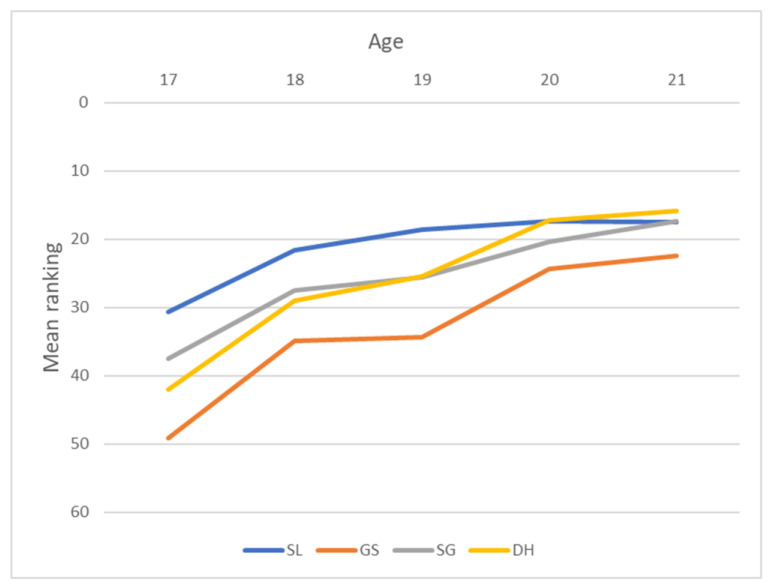
Increase of mean ranking across age category and events in female skiers. A lower ranking indicates better performance. (SL = slalom; GS = Giant slalom; SG = Super G; DH = downhill).

The performance, shown as mean rank, increased with age in all events, shown as a decrease in mean rank (Figure 2 and Table 8). The decrease in performance with age was larger from 17 years to 18 years than among the older age categories. The performance in terms of mean rank from 17 to 21 years increased more in downhill than in slalom for the female racers. Among female skiers, there were a few participants in the youngest age category, especially in speed events. The Jonckheere–Terpstra test for order alternatives showed a statistically significant trend (*p*-value < 0.005) of lower mean rank with age in female skiers in all events and the results are shown in Table 9.

## 4. Discussion

The aim of the present study was to investigate whether a constituent year effect (CYE) exists among skiers’ performance in the junior Alpine World Championships by examining whether the older skiers in the five-year cohort systematically outperform the younger skiers. Furthermore, it was investigated whether such an effect was more prominent among male skiers than female skiers. The third tested hypothesis was that the CYE would be more pronounced in speed events than in technical events.

### 4.1. The Overall Results

The results in the present study reveal a general CYE on both male and female skiers’ *performances* in the FIS Junior World Ski Championship in alpine skiing. The oldest skiers within the five-year age span (21-year-olds) perform overall better in all events compared to the younger ones in terms of mean ranking in the alpine race competitions. The increase in performances follows the ages neatly, with a significant linear trend within each event among both sexes.

### 4.2. Performance Measured as Completion of Races

In the junior World Championship, the competitors are assembled in a multi-year age band of 5 years. Bjerke, Lorås [21] showed that the participation rate increases with age, with an almost linear increase in competitors from 17-year-old to 21-year-old skiers. One measure of the performance of the skiers was the dichotomous variable completion of the race, or not completion, representing success, or no success. If a skier did not finish the race according to the rules, whatever the reason, it was considered a failure of performance. Reasons for failure could be falling, missing gates, hooking in a gate, or not being able to adjust the speed to the race conditions and skiing out of the course as a consequence.

The mean completion rate increased with increasing age. Across all events among males, a 17-year-old-skier was less likely to complete the race compared to a 20- or 21-year-old-skier, and the latter groups had a 1.79 and 1.9 times greater chance, respectively, of completing a race than the youngest group. The results imply that the older the racer is, the better the skills and prerequisites to finish the race by its slope conditions. Such skills may be technical, used for adjusting the speed to the slope and weather conditions, or psychologically adjusting the speed so that it matches the skier’s own skills [23]. No significant effects were found among female skiers.

The mean completion rate among males is higher in speed events than in the technical events, with the mean completion rate in slalom being 30%, whereas 92% of the skiers completed the races in downhill events. In alpine skiing, the speed events include downhill and super-G, whereas slalom and giant slalom constitute the technical events [24]. The latter two consist of two runs that are shorter in duration for each run, with an average speed of 54 and 65 km/h, respectively [25]. The speed events consist of one run with a longer duration, and they follow the gravity line of the hill, where the speed can reach a velocity of 120–130 km/h (the highest speed was measured as 161.9 km/h in Wengen) [26]. Making mistakes and falling at higher speed, obviously, has a greater impact on the racer in terms of injuries, and the injury rate in speed events seems to increase and to be related to increased speed and jumps [27]. Adjusting the technique during high-speed places great demands on the skier, and the tactical skills of the skier are of great importance for success in alpine ski racing [23]. In the present data, there was no significant difference in completion rate between events for any age category. The difference in completion rate is only found when comparing 17-year-old skiers with those aged 20 and 21, in all events, showing that skiers aged 17 years are skiing out more often than 20- or 21-year-old skiers. A possible explanation may be that there are too few skiers in each group for comparison when they are split by both event and age.

However, since the number of participants is significantly higher among the oldest racers, there are reasons to believe that the oldest skiers possess some skills and advantages that the youngest still do not have. The older skiers can adjust their skiing technique to the conditions, even at high speed, because of their superior strength and are thus able to make the appropriate tactical judgements required to succeed. An older skier has acquired more routine and experience than a younger one due to more days of skiing and variation in the training environment, which increases with age [25]. For example, skiers usually start practicing the speed events later than the technical events, and it takes years of specific practice to acquire sufficient skill and experience to be able to compete in an event. According to Musch and Grondin [1], both physical development and experience are factors that contribute to a relative age effect. A 17-year-old skier is not as physically developed as a 21-year-old one. According to Vermeulen, Clijsen [28], who investigated the event-specific body characteristics of elite alpine skiers, the muscle-to-fat ratio increases with age, and skiers competing in speed events are heavier than those in technical events. In a multi-year age band, being almost five years older has a significant impact on the athlete’s possibility to gain muscle weight through weight training and develop a physical somatotype advantageous for speed events. The maturation status aligns with previous findings on CYE in other individual sports, e.g., kayaking [29].

No effect of constituent year was found on performance measured as completion among female skiers, meaning that there is no significant difference between a 17-year-old skier and a 21-year-old skier in completing the race. In Bjerke, Lorås [21], a CYE was shown in terms of participation, and the effect lasted two years longer for male skiers than for female ones. They proposed as an explanation that this difference was due to puberty-related variables since the two-year difference coincided with the difference in the onset of puberty. Nevertheless, using the CYE as a proxy proves to be an appropriate way to study the effect of relative age between skiers because using multi-age cohorts allows a magnifying lens to be put on the RAE. The small effects that could be found within one year by studying the RAE were even more visible as CYEs. Of course, measuring participation cannot tell how well the skiers were actually performing, so in the present study, skiers’ rankings in each event were analysed in order to investigate how their performance actually varied with relative age (CYE) and event.

### 4.3. Performance as Results in Ranking

As was found by Bjerke, Lorås [21], when using participation in races as a proxy for performance (under the assumption that skiers and their coaches thoroughly assess the chances of success before deciding to participate), a clear CYE was found on performance as mean rank in races. These results are similar to those previously found in tennis ranking [30] and among young kayakers [29]. The mean ranking in races decreased with increasing age, thus indicating better performance or, more precisely, faster skiing with increasing age. The pattern of increasing success with increasing age was evident throughout all events and for both sexes. For example, 17-year-old male skiers in slalom were ranked, on average, as number 33, whereas the 21-year-olds, on average, occupied 15^th^ place. The same trend was evident in all events among men. The older the skier, the better the mean ranking in the final results. 

The results among female skiers are similar, showing decreasing rank (better performance) with age across all events. In speed events, and especially downhill, the results can be aligned with the number of skiers completing the race. In these events, there are just a few young skiers who have qualified for the JWC, and those who have completed are at the bottom of the results. These results can also be explained by the same arguments about developmental issues as for completion of the race. Over a five-year age span, these developmental differences between skiers are not just physical but due to experience, training history, and tactical decisions that are more pronounced in speed events than in technical events due to, for example, risks [23].

### 4.4. Implications

Both in terms of completion of the races and rankings in the races, there was a clear CYE, supporting the previous finding of a CYE on participation in races [21]. However, since the effect varied between events such that it increased with the increasing speed of the event, the explanation cannot be age alone. In fact, age may not even be the relevant variable, but rather some other variable affected by the speed of the event. This, logically, correlates closely with age; thus age is a proxy for that variable. The obvious candidate for that variable is development. Older skiers are not only more physically developed than younger skiers, but they are also more mature. Maturity reflects more experience and life history but also a longer training history. An older skier has, through strength training, as well as from growth alone, gained more weight and improved other physical characteristics [25]. Also, the older skier is more experienced in dealing with psychological challenges and making tactical considerations. These abilities become more visible in the speed events because the advantage of being stronger, heavier, and more experienced facilitates mastering the gravitational forces (heavy skiers are faster skiers) and being more experienced facilitates taking wise decisions in the tactical part as the speed increases. Thus, since the results vary with speed within the general CYE, our results imply that age affects speed; therefore, age is a proxy for development.

### 4.5. Methodological Limitations

There are methodological limitations in this study that warrant some further investigation. First, the online data from fis-ski.com only include information on skiers age, sex, and performance in the junior alpine World Championships. Thus, there are multiple psychological and physiological characteristics that influence the development of alpine skiers that are not included in the current study. E.g., previous studies have systematically connected physical capability and anthropometry to race performance in alpine skiing [31,32], and these measures have also been associated with the RAE across sports [33,34,35]. Skill development in alpine skiing is a multidimensional process, and further enquiries should examine, also at an individual level, what factors contribute to the CYE in junior elite alpine skiing.

Second, the FIS website does not contain any information regarding the shifting environmental conditions during the races, such as the structure of snow and slopes or weather conditions. In FIS competitions, the skiers usually start according to their awarded FIS points based on performance in previous races, which can disfavor younger and less experienced skiers that might end up competing later in races compared to the older and more experienced skiers. Thus, a late start in a race might impact performance due to shifting snow and weather conditions. However, this is an integrated part of high skill in alpine skiing since more experienced and skilled skiers can cope with challenging slope conditions by adapting the speed decision making to succeed in races [36].

A third limitation is the moderate sample size. In RAE studies, there are typically large sample sizes as trends and patterns are examined at an international sport level. However, the sample size needs to be evaluated against the specific population examined, which in the current study was junior elite alpine skiers (aged 17–21 years) participating in world championships. This population is not very large, and one might indeed argue that the present sample size represents a substantial proportion of this sub-population from the three seasons included in the data [37].

## 5. Conclusions

The present study examined whether a constituent year effect is evident among athletes in the Junior World Championship in alpine skiing. A previous study had suggested such an effect based on a proxy variable, but it lacked actual data on performance. The present results show, as was suggested, that older skiers systematically outperform younger skiers across all events. The effect is greater among male skiers but is also found in females. The CYE becomes increasingly stronger with the increasing speed of the events, indicating that age alone may not explain the difference. However, differences in development may also explain this effect. We argue that the CYE is more about differences in development than age. The ability to maintain control at higher speed increases as a result of physical development and experience; therefore, it correlates closely with age. Thus, the CYE is really a proxy for relative development. Consequently, the RAE, which the CYE is a magnified version of, is not a relative age effect but a development effect, and hence would be more correctly termed as the relative development effect (RDE).

## Figures and Tables

**Table 1:** Total number of participants separated by sex, age category, and event.

	All Events (N)		Slalom (N)		Giant Slalom (N)		Super G (N)		Downhill (N)	
Age	Male	Female	Male	Female	Male	Female	Male	Female	Male	Female
17	54	82	24	34	25	36	4	10	1	2
18	122	163	43	58	43	59	23	29	13	17
19	222	202	68	63	67	64	50	44	37	31
20	407	219	115	61	117	63	98	54	77	41
21	383	193	107	51	110	60	91	45	75	37
All age	1188	859	357	267	362	282	266	182	203	128

**Table 2:** Completed race for male skiers across events.

	All Events (N)			Slalom (N)			Giant Slalom (N)			Super G (N)			Downhill (N)		
Age	Started	Completed	%	Started	Completed	%	Started	Completed	%	Started	Completed	%	Started	Completed	%
17	54	23	42.6	24	6	25.0	25	14	56.0	4	2	50.0	1	1	100.0
18	122	65	53.3	43	10	23.3	43	25	58.1	23	18	78.3	13	12	92.3
19	222	126	56.8	68	24	35.3	67	30	44.8	50	38	76.0	37	34	91.9
20	407	232	57.0	115	36	31.3	117	59	50.4	98	65	66.3	77	71	92.2
21	383	224	58.5	107	31	29.0	110	56	50.9	91	70	76.9	75	69	92.0
All	1188	670	56.4	357	107	30.0	362	184	50.8	266	193	72.6	203	187	92.1

**Table 3:** Descriptive ORs for completing a race across all age cohorts separated for events for male skiers.

Gender	Disciplines	Statistic	17:18	17:19	17:20	17:21	18:19	18:20	18:21	19:20	19:21	20:21
Male	All events	OR	1.54	1.73	1.79	1.90	1.15	1.16	1.24	1.01	1.07	1.06
		(95% CI)	0.81–2.93	0.95–3.15	1.01–3.17	1.07–3.38	0.74–1.79	0.77–1.75	0.82–1.86	0.73–1.41	0.77–1.50	0.80–1.41
		*p*-value	0.19	0.08	0.05 *	0.03 *	0.53	0.47	0.31	0.95	0.68	0.67
	Slalom	OR	0.91	1.64	1.37	1.22	1.80	1.50	1.35	0.84	0.75	0.90
		(95% CI)	0.28–2.91	0.57–4.67	0.50–3.73	0.44–3.37	0.76–4.27	0.67–3.38	0.59–3.06	0.44–1.58	0.39–1.43	0.50–1.59
		*p*-value	0.87	0.36	0.54	0.70	0.18	0.32	0.48	0.58	0.38	0.70
	Giant Slalom	OR	1.09	0.64	0.79	0.81	0.58	0.73	0.76	1.25	1.28	1.02
		(95% CI)	0.40–2.95	0.25–1.61	0.34–1.91	0.34–1.95	0.27–1.27	0.36–1.48	0.37–1.52	0.69–2.29	0.70–2.35	0.61–1.72
		*p*-value	0.86	0.34	0.61	0.65	0.17	0.39	0.42	0.46	0.43	0.94
	Super G	OR	- **	-	-	-	0.88	0.55	0.93	0.62	1.05	1.69
		(95% CI)					0.27–2.88	0.19–1.60	0.31–2.79	0.29–1.35	0.47–2.37	0.89–3.21
		*p*-value					0.83	0.27	0.89	0.23	0.90	0.11
	Downhill	OR	-	-	-	-	-	-	-	-	-	-
		(95% CI)										
		*p*-value										

OR: Odds Ratio. CI: Confidence Interval. * = Significant at level *p* < 0.05; - = too few participants for calculating ORs. ** = ORs were not calculated due to few participating skiers.

**Table 4:** Mean ranking in the male races across age and events, including standard deviation (SD) and the number of skiers completing the races.

Event	Age	17	18	19	20	21
Slalom	Rank	33.0	23.0	20.6	16.6	15.2
	SD	7.32	10.47	10.5	10.21	9.83
	N	6	10	24	36	31
Giant slalom	Rank	49.8	44.4	32.6	32.8	21.6
	SD	14.01	18.5	19.56	18.51	15.78
	N	14	25	30	59	56
Super G	Rank	50.5	49.1	39.4	30.3	26.4
	SD	16.26	15.9	18.67	17.28	17.09
	N	2	18	38	65	70
Downhill	Rank	54.0	46.3	37.2	30.1	27.1
	SD	-	16.01	15.29	16.96	19.20
	N	1	12	34	71	67

**Table 5:** The *p*-values of lower mean rank with age in male skiers from the Jonckheere–Terpstra test.

Event	TJT *	z-Value	*p*-Value
Slalom	178.4	−3.56	0.001
Giant Slalom	402.8	−5.81	0.001
Super G	241.9	−4.94	0.001
Downhill	229.3	−2.88	0.001

* TJT = the value of the Jonckheere–Terpstra test statistics.

**Table 6:** Completed race for female skiers across events.

	All Events (N)			Slalom (N)			Giant Slalom (N)			Super G (N)			Downhill (N)		
Age	Started	Completed	%	Started	Completed	%	Started	Completed	%	Started	Completed	%	Started	Completed	%
17	85	48	58.5	34	14	41.2	36	23	63.9	10	9	90.0	2	2	100.0
18	163	101	62.0	58	25	43.1	59	44	74.6	29	16	55.2	17	16	94.1
19	202	134	66.3	63	32	50.8	64	41	64.1	44	32	72.7	31	29	93.5
20	219	137	62.6	61	24	39.3	63	38	60.3	54	39	72.2	41	36	87.8
21	193	128	66.3	51	23	45.1	60	37	61.7	45	31	68.9	37	37	100.0
All	859	548	63.8	267	118	44.2	282	183	64.9	182	127	69.8	128	120	93.8

**Table 7:** Descriptive ORs for completing a race across all age cohorts separated for events for female skiers.

Gender	Disciplines	Statistic	17:18	17:19	17:20	17:21	18:19	18:20	18:21	19:20	19:21	20:21
Female	All events	OR	1.15	1.4	1.18	1.39	1.21	1.03	1.21	0.85	0.85	1.18
		(95% CI)	0.67–1.98	0.82–2.37	0.71–1.99	0.82–2.37	0.79–1.86	0.68–1.56	0.78–1.87	0.57–1.27	0.57–1.26	0.79–1.77
		*p*-value	0.60	0.21	0.52	0.21	0.39	0.91	0.39	0.42	0.42	0.43
Slalom		OR	1.08	1.47	0.93	1.17	1.36	0.86	1.08	0.63	0.80	1.27
		(95% CI)	0.46–2.55	0.63–3.43	0.40–2.18	0.49–2.82	0.66–2.79	0.41–1.78	0.51–2.31	0.31–1.28	0.38–1.67	0.60–2.69
		*p*-value	0.86	0.37	0.86	0.72	0.40	0.68	0.83	0.20	0.55	0.54
Giant Slalom		OR	1.66	1.01	0.86	0.91	0.61	0.51	0.55	0.85	0.90	1.05
		(95% CI)	0.68–4.07	0.43–2.35	0.37–2.00	0.38–2.14	0.27–1.32	0.23–1.12	0.25–1.20	0.41–1.75	0.43–1.87	0.51–2.19
		*p*-value	0.27	0.99	0.73	0.83	0.21	0.10	0.13	0.85	0.78	0.88
Super G		OR	-	-	-	-	2.17	2.11	1.80	0.97	0.83	0.85
		(95% CI)					0.81–5.82	0.82–5.43	0.68–4.78	0.40–2.38	0.33–2.07	0.35–2.01
		*p*-value					0.12	0.12	0.23	0.96	0.69	0.72
Downhill		OR	-	-	-	-	-	-	-	-	-	-
		(95% CI)										
		*p*-value										

OR: Odds Ratio. CI: Confidence Interval; - = too few participants for calculating ORs.

**Table 8:** Mean ranking in the female races across age and events, including standard deviation (SD) and the number of skiers completing the races.

	Age	17	18	19	20	21
Slalom	Rank	30.7	21.6	18.6	17.4	17.5
	SD	6.68	11.46	11.16	11.04	11.67
	N	14	25	32	24	23
Giant slalom	Rank	49.1	34.9	34.3	24.3	22.4
	SD	15.45	19.69	15.21	18.71	15.38
	N	23	44	41	38	37
Super G	Rank	37.6	27.4	25.6	20.4	17.4
	SD	14.05	15.17	13.33	14.71	11.08
	N	9	16	32	39	31
Downhill	Rank	42.0	29.1	25.5	17.3	15.9
	SD	4.24	11.67	9.72	12.13	10.51
	N	2	21.6	29	36	37

**Table 9:** The *p*-values of lower mean rank with age in female skiers from the Jonckheere–Terpstra test.

Event	TJT *	z-Value	*p*-Value
Slalom	209.6	−3.15	<0.001
Giant Slalom	404.6	−5.65	<0.001
Super G	232.1	−3.75	<0.001
Downhill	211.9	−4.71	<0.001

* TJT = the Jonckheere–Terpstra test.

## Data Availability

The data are publicly available online and could be found at the FIS web site: (https://www.fis-ski.com/DB/general/statistics.html?sectorcode=AL).
